# The peripheral blood mononuclear cells versus purified CD34^+^ cells transplantation in patients with angiitis-induced critical limb ischemia trial: 5-year outcomes and return to work analysis—a randomized single-blinded non-inferiority trial

**DOI:** 10.1186/s13287-022-02804-4

**Published:** 2022-03-21

**Authors:** Hao Liu, Tianyue Pan, Yifan Liu, Yuan Fang, Gang Fang, Xiaolang Jiang, Bin Chen, Zheng Wei, Shiyang Gu, Peng Liu, Weiguo Fu, Zhihui Dong

**Affiliations:** 1 Departments of Vascular Surgery of Zhongshan Hospital, Fudan University, 180 Fenglin Road, Shanghai, 200032 China; 2 Departments of Hematology of Zhongshan Hospital, Fudan University, Shanghai, China; 3 National Clinical Research Center for Interventional Medicine, Shanghai, China

**Keywords:** Critical limb ischemia, Cell transplantation, CD34^+^

## Abstract

**Backgrounds:**

Patients with AICLI constitute a considerable proportion of NO-CLI patients and cannot be treated with surgical or endovascular treatment. Although cell therapy has shown satisfactory results in treating AICLI, research comparing the efficacy of treatment with the 2 kinds of cell products is rare. The aim of this study was to report the 5-year outcomes of a randomized single-blinded noninferiority trial (Number: NCT 02089828) on peripheral blood mononuclear cells (PBMNCs) and purified CD34^+^ cells (PCCs) transplantation for treating angiitis-induced critical limb ischemia (AICLI).

**Methods:**

A randomized single-blinded non-inferiority trial (Number: NCT 02089828) was performed. Fifty patients were randomized 1:1 to the PBMNCs and PCCs groups. Efficacy outcomes, safety outcomes and patients’ work conditions were analyzed. The primary efficacy outcomes included major amputation and total amputation over 60 months.

**Results:**

During the 60-month follow-up, 1 patient was lost to follow-up, 1 died, and 2 underwent major amputation. The major amputation-free survival rate (MAFS) was 92.0% (95% confidence interval [CI] 82.0%-100.0%) in the PBMNCs group and 91.7% (95% CI 81.3%-100.0%) in the PCCs group (*P* = 0.980). Compared with the PCCs group, the PBMNCs group had a significantly higher 5-year new lesion-free survival rate (100.0% vs. 83.3% [95% CI 69.7–99.7%], *P* = 0.039). All patients lost their ability to work before transplantation, and the 5-year cumulative return to work (RTW) rates were 88.0% in the PBMNCs group and 76.0% in the PCCs group (*P* = 0.085).

**Conclusion:**

The long-term follow-up outcomes of this trial not only demonstrated similar efficacy and safety for the 2 types of autoimplants but also showed a satisfactory cumulative RTW rate in AICLI patients who underwent cell transplantation.

*Trial registration*: ClinicalTrials.gov, number NCT 02089828. Registered 14 March 2014, https://clinicaltrials.gov/ct2/show/record/NCT02089828.

## Introduction

No-option critical limb ischemia (NO-CLI) is defined as ischemia that cannot be treated with surgical or endovascular treatment owing to a high postoperative reocclusion rate and poor anatomical conditions [1–3]. Patients with angiitis-induced critical limb ischemia (AICLI), which is defined as ischemia caused by thromboangiitis obliterans (TAO) or other arteritis-related autoimmunological diseases, such as systemic lupus erythematosus (SLE), psoriasis, or Crohn's disease, constitutes a considerable proportion of NO-CLI patients. AICLI patients, taking TAO patients as an example, are usually relatively young and have a high amputation rate; thus, they pose a heavy burden to family and society [4].

Cell therapy, a promising treatment method including peripheral blood mononuclear cells (PBMNCs) and purified CD34^+^ cells (PCCs) transplantation, has shown satisfactory results in treating NO-CLI patients [5, 6]. Although endothelial progenitor cells (EPCs) are the main effector cells, the 2 kinds of autoimplants are characterized by different compositions and proportions of cells. However, research comparing the efficacy of the 2 kinds of cell products is rare. In 2014, we launched the first clinical trial (NCT 02089828) specifically designed to evaluate the therapeutic efficacy of PBMNCs versus PCCs transplantation in the treatment of AICLI. The 1-year and 3-year outcomes were reported previously [7, 8], and except for earlier pain relief and less pain at the injection sites in the PCCs group and earlier ischemia improvement in the PBMNCs group, equivalent efficacy was observed between the two types of autoimplants. The current study aims to compare the long-term outcomes of this trial and to report patients’ postoperative work conditions, given that almost all patients were unable to work due to severe pain and/or foot ulcers and that they were the main breadwinners in their families.

## Methods

### Study design

From April 2014 to September 2021, 50 patients were enrolled in this trial. This trial was a prospective, single-center, single-blinded, randomized noninferiority trial of PBMNCs versus PCCs for the treatment of patients with AICLI and Rutherford class 4 to 5 status. Details of the trial design were described previously [7, 8].

All the participants signed informed consent forms before enrollment. This study was conducted in compliance with the Declaration of Helsinki and other applicable regulatory requirements. This study was approved by the Ethics Committee of Fudan University Affiliated Zhongshan Hospital. This trial was registered with ClinicalTrials.gov, number NCT 02089828.

### Patients

Details of the inclusion and exclusion criteria were described previously [7, 8]. The eligible patients were allocated in equal proportion to the PBMNCs or PCCs group at random using a computer-generated randomization schedule (SAS, Proc Mixed, version 8.2).

### Randomization and masking

All of the patients were masked during hospitalization. The cell transplantations, the independent decisions about minor or major amputations after the transplantations and the patients' follow-up data collection, assessments and analysis were completed by 3 different groups of surgeons who were blinded to the other groups’ work. Blinding was removed if a serious adverse event occurred that was related to the trial, if death or loss to follow-up occurred or if an emergency required unblinding.

### Procedures

RhG-CSF (Neupogen®; Amgen, Thousand Oaks, CA, USA) (5–10 μg/kg per day) and enoxaparin (4000 IU/day) were administered subcutaneously to all patients for 4 days. On the 5th day, apheresis (COM. TEC; Fresenius Hemocare GmbH, Bad Homburg, Germany) was performed. For the patients in the PBMNCs group, cells separated by apheresis were washed 3 times and resuspended in an ethylenediaminetetraacetic acid-phosphate buffered saline solution (200 mL) that contained 0.5% human albumin. For the patients in the PCCs group, CD34^+^ cells were purified using a magnetic cell sorting system (Miltenyi Biotec GmbH, Bergisch Gladbach, Germany) immediately after leukapheresis. The final cell products were assessed by flow cytometry and leukocyte counting. With patients under general anesthesia, the surgeons implanted the cells into the calves/arms and feet/hands of the ischemic limbs via equidistant intramuscular injections (0.5 mL/site). Additional details about the procedure were described previously [7].

### Efficacy and safety outcomes

Patients were followed for a total of 60 months and had clinical visit evaluations according to the following schedule: 1, 2, 3, 6, 12, 24, 36, 48 and 60 months. The primary efficacy outcome over 60 months was amputation, including major amputation and minor amputation. Major amputation was defined as amputation above the ankle, and minor amputation was defined as below the ankle. Other efficacy outcomes included the Wong–Baker Faces Pain Rating Scale (WBFPS; a score of 0 represents no pain and a score of 10 represents the greatest pain) score, Rutherford classification, pain-free walking time (PFWT; at 2.5 km/h and at a 10% incline on a treadmill), ankle-brachial index (ABI), toe-brachial index (TBI), transcutaneous oxygen pressure (TcPO_2_), quality of life (QoL), recurrence (transplanted limb in AICLI condition again), new lesions (untransplanted limb in AICLI condition) and return to work (RTW) [7, 9, 10]. RTW was defined as full-time or part-time employment after transplantation, and work condition was classified as return to the same work, change in work, sick leave and normal retirement. Safety outcomes included all-cause mortality, all adverse events from mobilization to 2 weeks after injection, pathological retinal angiogenesis and sustained elevation of leukocyte counts. During the follow-up, any patient who was lost or dead was considered a worst-case scenario.

### Statistical analyses

The quantitative data are shown as the estimated margin mean (EMM) ± standard error (SE) (for comparison between 2 groups), median with interquartile range (IQRs) or mean ± standard deviation (SD), depending on their distribution. Categorical data are presented as numbers with percentages. Pearson's Chi-squared test with or without Yete's continuity correction or Fisher's exact test was used to compare all-cause mortality, RC and recurrence/new lesions between the groups. Kaplan–Meier curves and the Breslow-Wilcoxon test were used to depict and compare the MAFS and TAFS. The significance level was set at 0.05 for all statistical tests. A linear mixed model was used to analyze the effects of cell type on the longitudinal changes in the continuous variables and to determine the presence of any interactions between the individual groups and the time point. The Wilcoxon signed-rank test was used to compare the QoL scores at baseline and at 1 year, 3 years and 5 years posttransplantation. All tests were performed using PASW software, version 19 (IBM Corporation, Armonk, NY, USA).

### Role of funding source

The funder of the study had no role in study design, data collection, data analysis, data interpretation, or writing of the report.

## Results

**Fig. 1:**
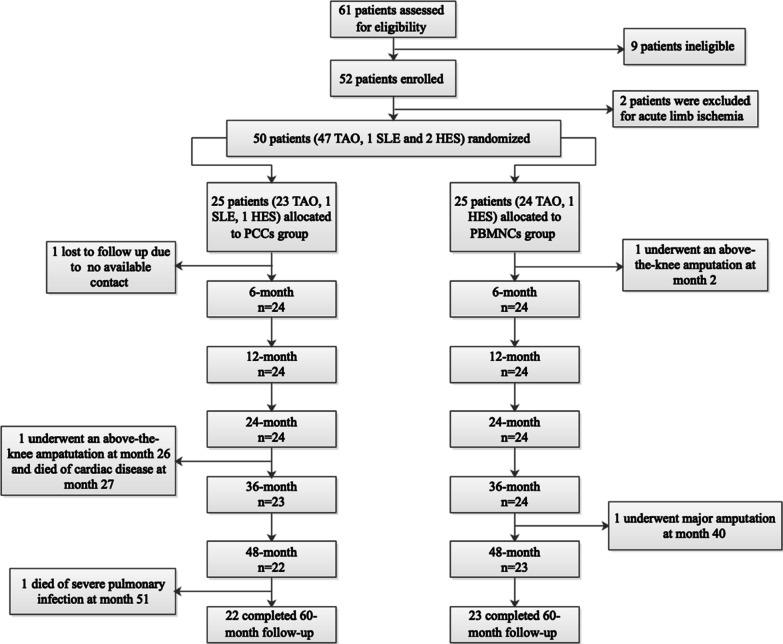
Protocol of the study. *Legend*: TAO, thromboangiitis obliterans; SLE, systemic lupus erythematosus; HES, hypereosinophilic syndrome; PBMNCs, peripheral blood mononuclear cells; PCCs, purified CD34^+^ cells

From April 2014 to September 2021, 50 of 61 AICLI patients were randomized to receive PBMNCs (*n* = 25) or PCCs (*n* = 25) transplantation. One patient was lost to follow-up (PCCs group), 1 patient (PBMNCs group) underwent a major amputation within 6 months after transplantation, 1 patient (PCCs group) underwent a major amputation at 26 months and died of cardiac disease at 27 months, and another patient (PCCs group) died of severe pulmonary infection at 41 months. The remaining 46 patients completed the 60-month follow-up (Fig. 1). The baseline characteristics of the patients are detailed in our previous study [7]. Briefly, the enrolled patients were all males with AICLI, with a mean age of 41.46 years, and were at low risk for cardiovascular and cerebrovascular disease risk factors, and most patients had a history of smoking (84% in the PCCs group and 92% in the PBMNCs group). Forty-seven patients had TAO, 1 patient had SLE, and 2 patients had hypereosinophilic syndrome (HES). The preoperative RC, ABI, TBI, and TcPO_2_ values were similar between the two groups. All patients were working before the onset of AICLI and lost their ability to work at admission. No significant differences were observed between the groups in any of the baseline characteristics.

Safety outcomes over 60 months are reported in Table 1. During hospitalization, no adverse events, including death, cardio-cerebrovascular events, and hepatic or renal dysfunction, occurred. Perioperative pain at the injection site was more frequently observed in the PBMNCs group (14/25 vs. 2/25, *P* < 0.001). During the follow-up, as mentioned above, 2 PCCs patients died, and no adverse events, including pathological retinal angiogenesis or tumorigenesis, were observed.

**Table 1:** Safety Outcomes of All Patients through 60 months based on worst-case scenarios

	PBMNCs group **(***n* = 25**)**	PCCs group **(***n* = 25**)**	*P* value
Mobilization-related adverse events^*^, *n* (%)	5 (20.0)	7 (28.0)	0.508
Pain at injection site, *n* (%)	14 (56.0)	2 (8.0)	< 0.001
Elevation of leukocyte counts > 3 days after transplantation, *n* (%)	1 (4.0)	2 (8.0)	0.552
All-cause mortality, *n* (%)	0 (0.0)	3 (12.0)^†^	0.074
Cardiovascular events, *n* (%)	0 (0.0)	1 (4.0)	0.312
Cerebrovascular events, *n* (%)	0 (0.0)	2 (8.0)	0.149
Pathological retinal angiogenesis, *n* (%)	0 (0)	0 (0.0)	1.000

The data presented are the numbers (%)

PBMNCs, peripheral blood mononuclear cells; PCCs, purified CD34^+^ cells

*Mobilization-related adverse events included slight fevers, transient headaches, back pains, and pruritus

^†^A lost patient was calculated based on worst-case scenarios

Except for 2 PCCs patients who received autoimplants with a CD34^+^ cell dose of < 10^5^/kg (3.54 × 10^4^ cells/kg and 4.56 × 10^4^ cells/kg, respectively), the patients all received cell transplants comprising a CD34^+^ cell dose of no < 10^5^/kg. Compared with PCCs, PBMNCs were characterized by a larger transplantation volume (80 mL [IQR: 60–110 mL] vs. 39 mL [IQR: 38–40 mL], *P* < 0.001) and higher total WBC count (25,800 × 10^6^ [15,200–44,100 × 10^6^] vs. 54.8 × 10^6^ [34.7–89.9 × 10^6^], *P* < 0.001). The CD34^+^ cell concentration did not differ significantly between the 2 groups (PBMNCs vs. PCCs, 8.61 × 10^8^/L [4.35–21.6 × 10^8^/L] vs. 8.00 × 10^8^/L [3.96–9.92 × 10^8^/L], *P* = 0.662).

**Fig. 2:**
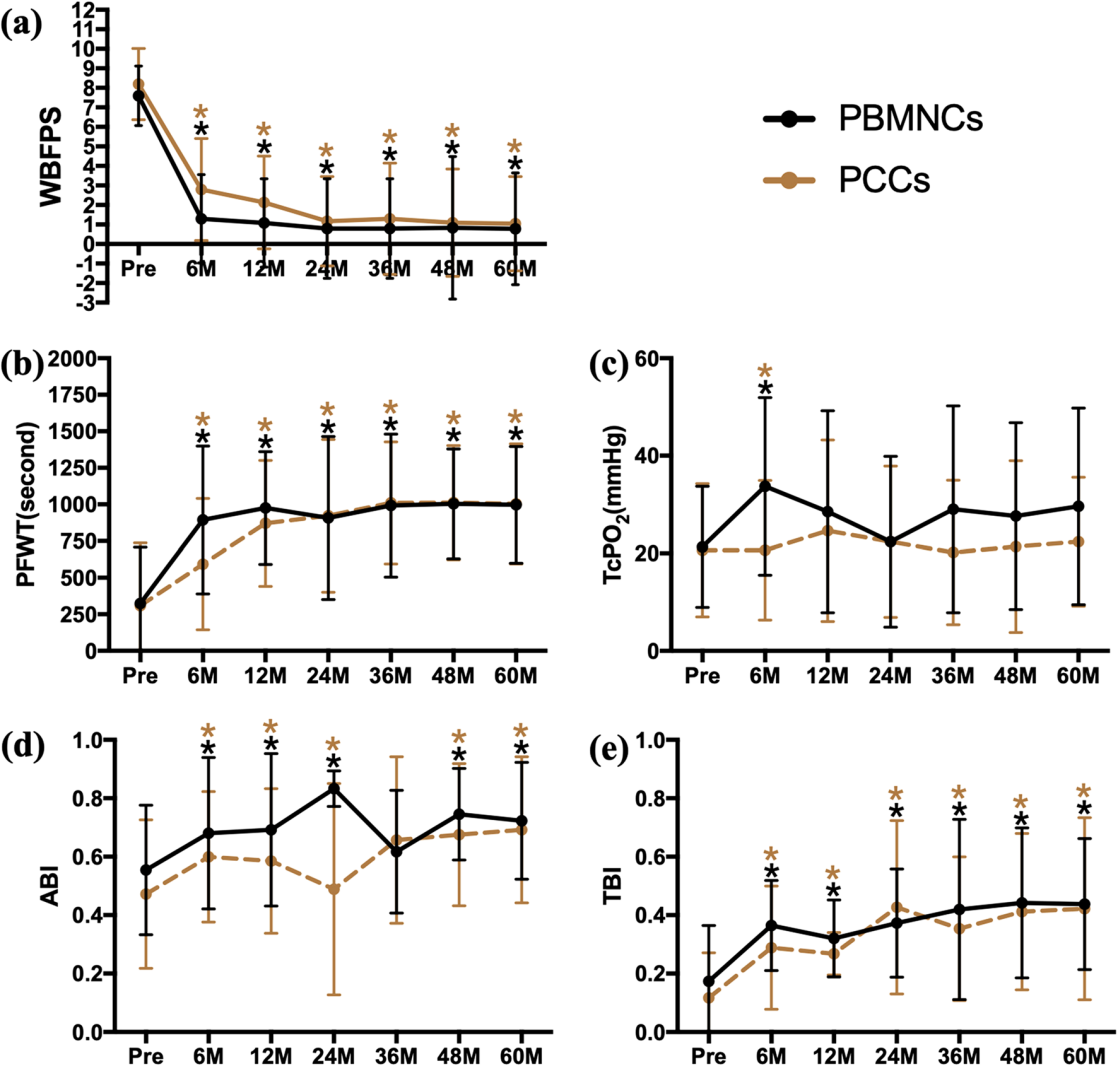
Longitudinal changes in pain relief, functional improvement and blood perfusion restoration. *Legend*: The assessments of pain were accessed based on **a** the Wong–Baker Faces Pain Rating Scale, the functional improvement was assessed based on **b** the pain-free walking time, and blood perfusion restoration was assessed with **c** the transcutaneous oxygen pressure, **d** the ankle-brachial index, and e, the toe-brachial index. The values are presented in linear graphs that show the means and SDs. * P < .05 vs. baseline; ** P < .01 vs. baseline. WBFPS, Wong–Baker Faces Pain Rating Scale; PFWT, pain-free walking time; TcPO_2_, transcutaneous oxygen pressure; ABI, ankle-brachial index; TBI, toe-brachial index; PBMNCs, peripheral blood mononuclear cells; PCCs, purified CD34^+^ cells

During the 5-year follow-up, significant improvements in WBFPS, PFWT and TBI values were observed in both groups 6 months after transplantation and sustained up to 60 months (Fig. 2A, B, F). Regarding TcPO_2_, significant improvements were only observed 6 months after transplantation and were observed in both groups (Fig. 2C). In both groups, significant improvements in ABI were observed at most timepoints during the 5-year follow-up, except at 36 months (Fig. 2D). No significant difference was observed between the 2 groups in terms of the outcomes mentioned above at any timepoint (Table 2).

**Table 2:** Efficacy outcomes of WBFPS, PFWT, TcPO_2_, ABI and TBI over time

	Group	Baseline	*P* value	1 year	*P* value	3 years	*P* value	5 years	*P* value
WBFPS	PBMNCs	7.60 ± 1.53	0.503	1.08 ± 2.26	0.116	0.79 ± 2.55	0.508	0.78 ± 2.86	0.730
	PCCs	7.28 ± 1.81		2.13 ± 2.38		1.30 ± 2.85		1.04 ± 2.42	
PFWT, seconds	PBMNCs	323.7 ± 383.8	0.898	976.1 ± 385.1	0.365	992.7 ± 488.4	0.894	998.2 ± 398.6	0.956
	PCCs	308.8 ± 430.3		870.6 ± 429.8		1010.0 ± 417.4		1004.6 ± 410.3	
TcPO_2_, mmHg	PBMNCs	21.3 ± 12.4	0.851	28.5 ± 20.7	0.487	29.0 ± 21.2	0.095	29.6 ± 20.2	0.142
	PCCs	20.6 ± 13.7		24.6 ± 18.6		20.2 ± 14.8		22.4 ± 13.2	
ABI	PBMNCs	0.554 ± 0.222	0.230	0.692 ± 0.261	0.147	0.617 ± 0.210	0.575	0.723 ± 0.200	0.631
	PCCs	0.472 ± 0.254		0.586 ± 0.247		0.657 ± 0.285		0.692 ± 0.250	
TBI	PBMNCs	0.174 ± 0.191	0.252	0.321 ± 0.132	0.085	0.420 ± 0.308	0.407	0.438 ± 0.224	0.836
	PCCs	0.117 ± 0.155		0.268 ± 0.073		0.354 ± 0.246		0.422 ± 0.312	

The data are presented as mean ± SD

WBFPS, Wong–Baker Faces Pain Rating Scales; PFWT, pain-free walking time; TcPO_2_, transcutaneous oxygen pressure; ABI, ankle-brachial index; TBI, toe-brachial index; PBMNC, peripheral blood mononuclear cell; PCC, purified CD34^+^ cell; SD, standard deviation

**Fig. 3:**
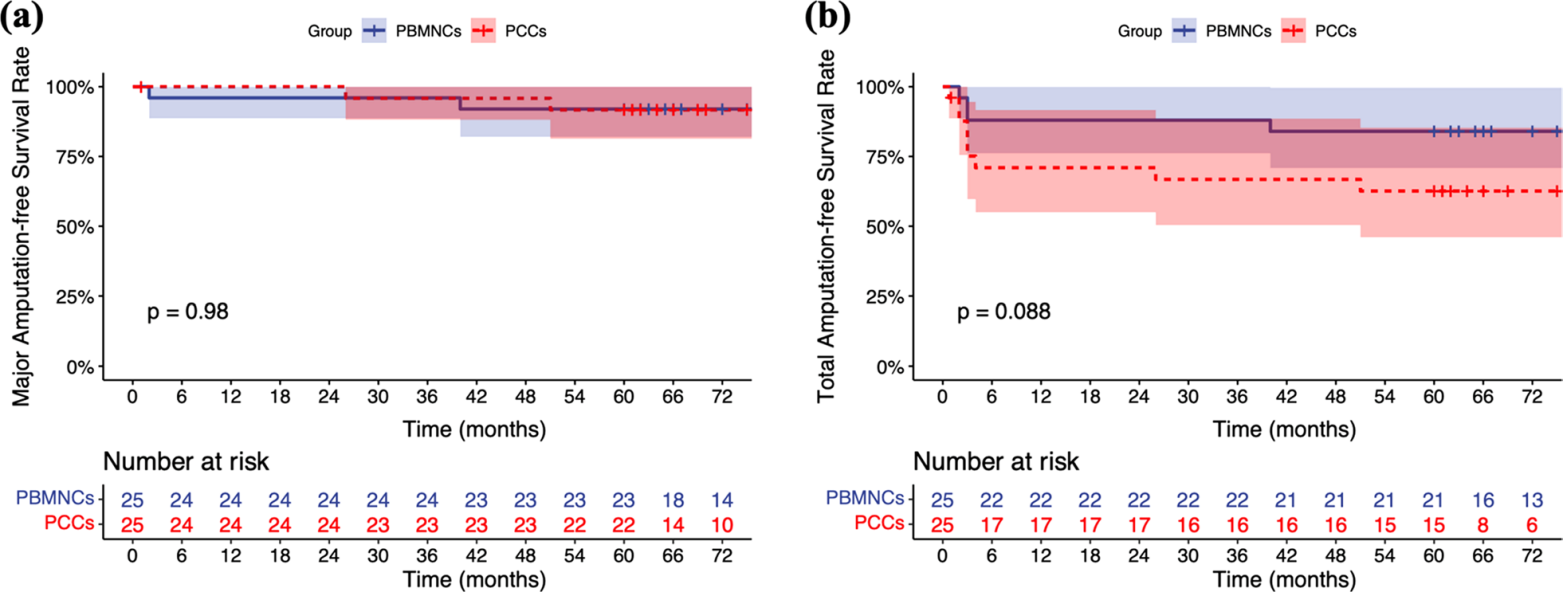
Kaplan–Meier curves showing the probabilities of a, major amputation-free survival and b, total amputation-free survival in both groups. *Legend*: PBMNCs, peripheral blood mononuclear cells; PCCs, purified CD34^+^ cells

During transplantation, 2 patients underwent simultaneous debridement due to severe ulcer/gangrene infection. During the follow-up, major amputation was observed in 1 PCCs patient at 27 months and in 2 PBMNCs patients at 2 months and 40 months. At 5 years, the MAFS was 92.0% (95% confidence interval [CI] 82.0–100.0%) in the PBMNCs group and 91.7% (95% CI 81.3–100.0%) in the PCCs group. Minor amputation was performed in 9 patients (3 in PBMNCs and 6 in PCCs) within 6 months. The 5-year TAFS was 84.0% (95% CI 70.8–99.7%) in the PBMNCs group and 62.6% (95% CI 46.0–85.3%) in the PCCs group. The 2 groups did not differ with respect to the probability of MAFS (log rank test: *P* = 0.980) or TAFS (log rank test: *P* = 0.088) (Fig. 3).

**Fig. 4:**
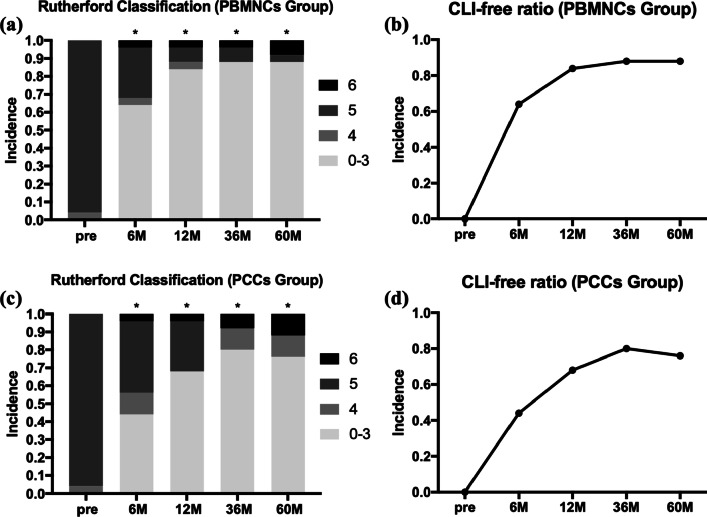
The change in Rutherford classification and CLI-free ratio during the 5-year follow-up. *Legend*: Serial changes in Rutherford classification (0–6) proportions of the **a** PBMNCs and **c** PCCs groups and serial changes in the CLI-free ratio of the **b** PBMNCs and **d** PCCs groups. ^*^ P < .05 vs. baseline; ^**^ P < .01 vs. baseline. PBMNCs, peripheral blood mononuclear cells; PCCs, purified CD34^+^ cells; CLI, critical limb ischemia

As we mentioned above, all patients were enrolled with RC 4–5 status (1 PBMNCs patient in RC 4, 1 PCCs patient in RC 4, 48 patients in RC 5). As we reported previously [8], the Rutherford classification in the PBMNCs group improved significantly by 3 months (*P* < 0.05) and 6 months (*P* < 0.001) in the PCCs group. During the 60-month follow-up, the improvement was sustained for up to 60 months, with 88.0% of PBMNCs recipients and 76.0% of PCCs recipients being CLI-free 5 years after transplantation (Fig. 4).

**Fig. 5:**
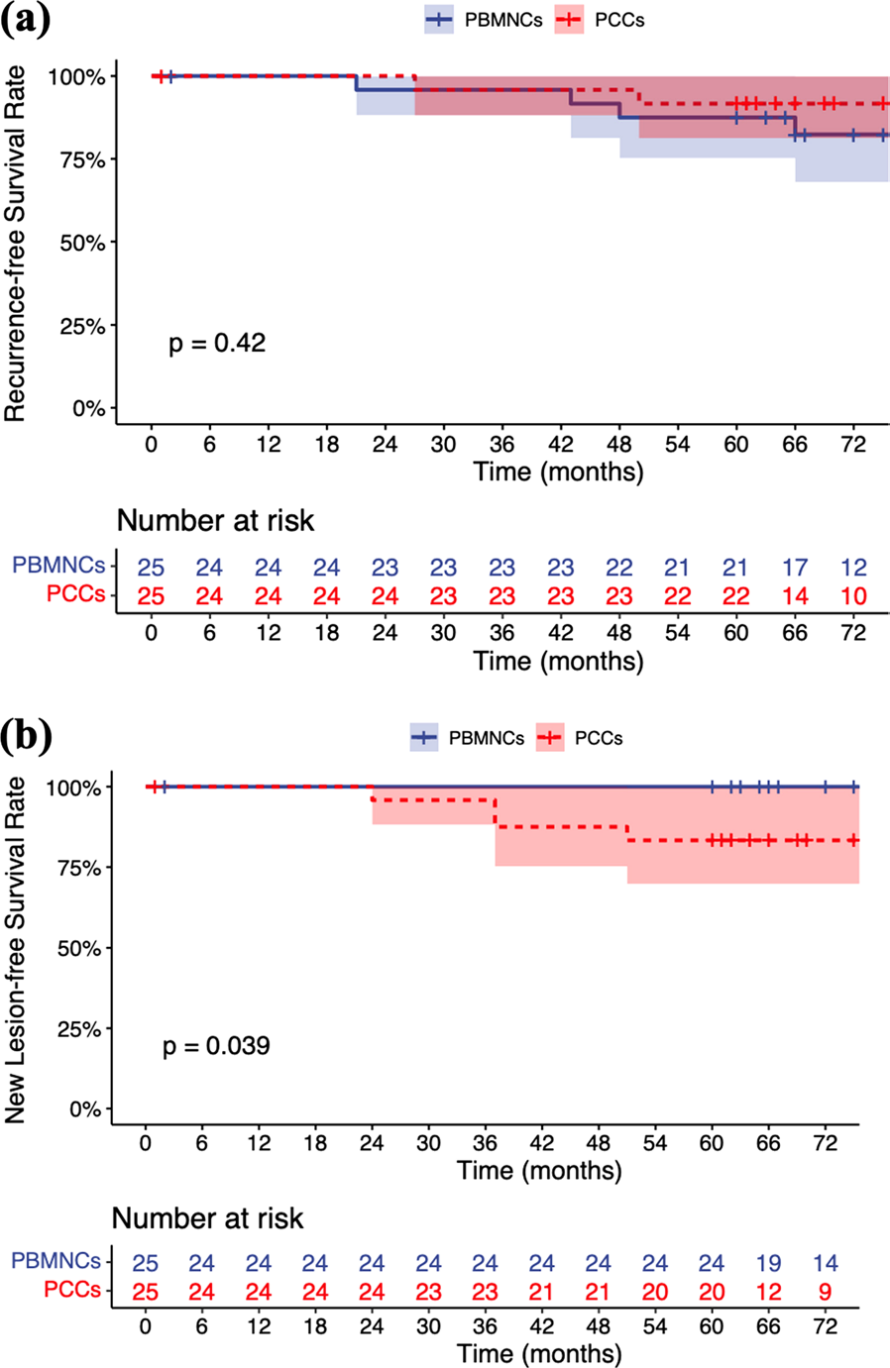
Title: Kaplan–Meier curves showing the probabilities of **a** recurrence-free survival and **b** new lesion-free survival in both groups. *Legend*: PBMNCs, peripheral blood mononuclear cells; PCCs, purified CD34^+^ cells

During the whole follow-up period, recurrence was observed in 5 patients. Three PBMNCs patients’ transplanted limbs returned to RC 4 status, and 2 patients’ (1 in each group) transplanted limbs had recurrent ulcers. Recurrent resting pain was relieved in 2 PBMNCs patients with conservative treatment, including antiplatelet drugs, vasodilators and exercise. One PBMNCs patient with unrelieved recurrent resting pain underwent a second cell transplantation 71 months after the first transplantation, 1 PCCs patient with unrelieved recurrent ulcers died of pulmonary infection 1 month after recurrence (50 months), and 1 PBMNCs patient with unrelieved recurrent gangrene underwent below-knee amputation at 40 months due to rapid ischemia progression and severe infection. The 5-year recurrence-free survival rates were 87.5% (95% CI 75.2%-100.0%) in the PBMNCs group and 91.7% (95% CI 81.3%-100.0%) in the PCCs group (log rank test: *P* = 0.416) (Fig. 5A).

New lesions occurred in 3 PCCs patients, including 1 with resting pain in the right lower limb and 2 with gangrene in the left lower limb. Two of them (1 with RC 4 status and 1 with RC 5 status) underwent retransplantation after adequate conservative treatment for at least 1 month, and their symptoms were all relieved within 6 months after the second transplantation. The remaining 1 patient underwent major amputation due to rapid progression of the ischemia at 26 months and ultimately died of cardiac disease at 27 months. Compared with PCCs patients, PBMNCs patients seemed more likely to survive to the 5-year follow-up without developing new lesions, with a significantly higher 5-year new lesion-free survival rate (100.0% vs. 83.3% [95% CI 69.7%-99.7%], log rank test: *P* = 0.039) (Fig. 5B). All recurrence/new lesion events occurred after the 12-month follow-up, with a mean recurrence/new lesion time of 40.8 ± 14.5 months (range 21–66 months).

**Fig. 6:**
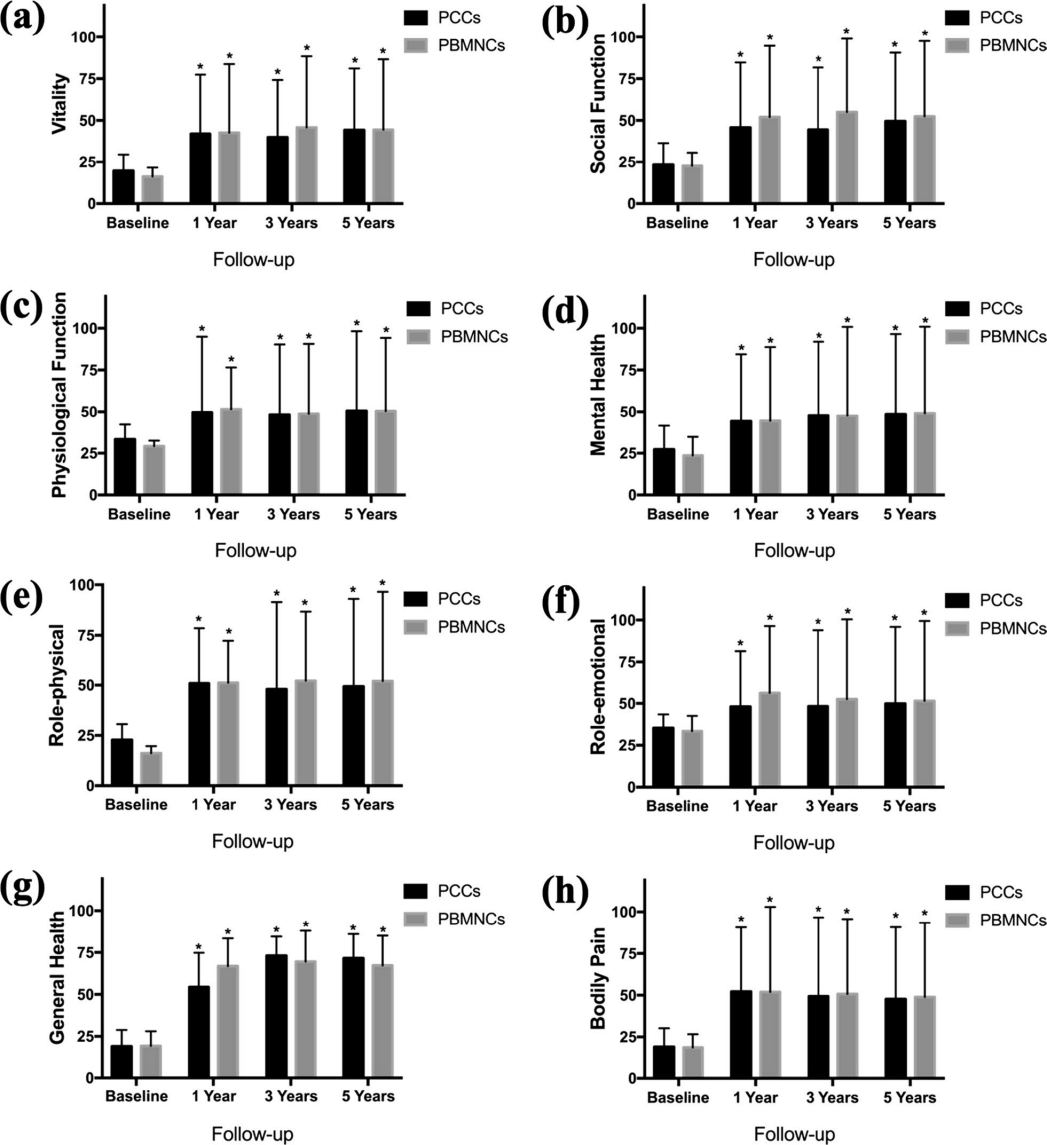
Quality of life at baseline and at 1 year, 3 years and 5 years after transplantation. *Legend*: Quality of life was assessed using the Short Form-36 (SF-36) scoring system (version 2) in the PCCs and PBMNCs groups. The SF-36 examines eight domains: **a** vitality, **b** social function, **c** physiological function, **d** mental health, **e** role-emotional, **f** role-physical **g** general health, and **h** bodily pain. * P < .05 (intragroup comparison with baseline, based on the Wilcoxon signed-rank test). PBMNCs, peripheral blood mononuclear cells; PCCs, purified CD34^+^ cells

**Fig. 7:**
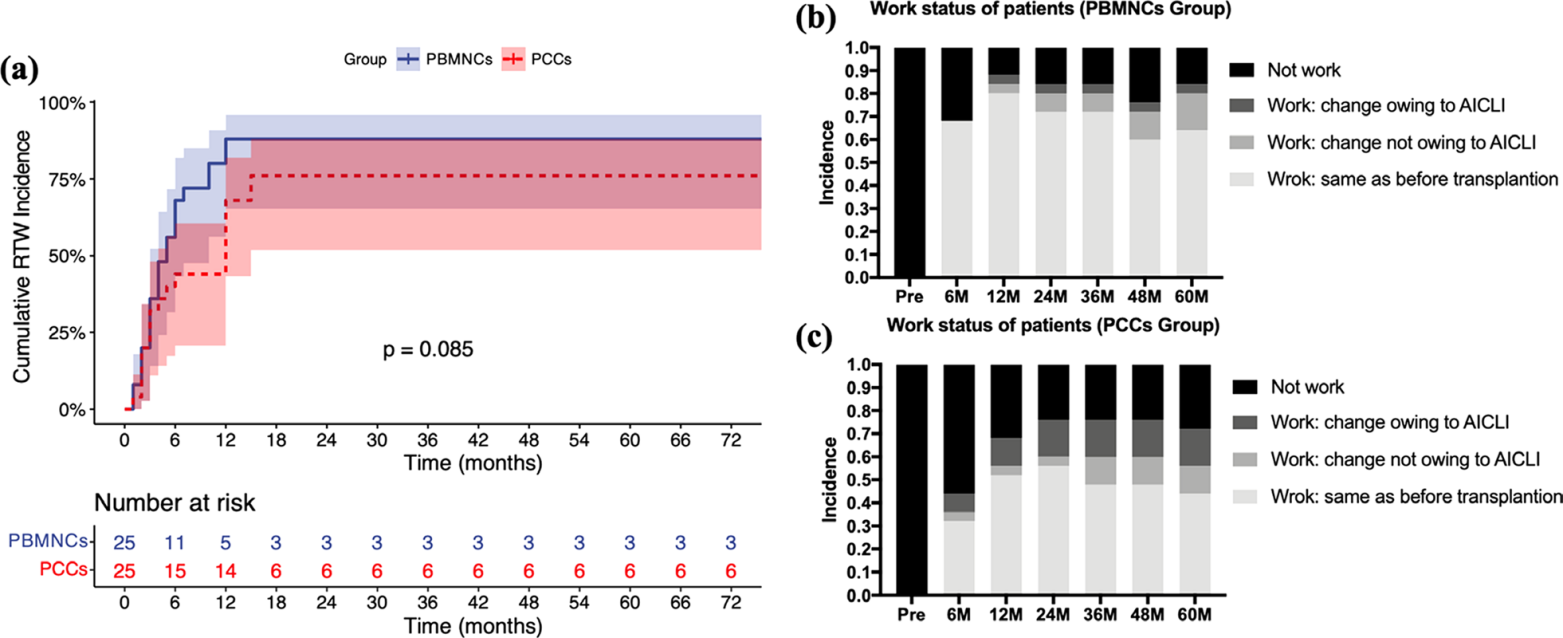
Kaplan–Meier curves showing the probabilities of **a** cumulative RTW incidence in both groups and serial changes in work status proportions in the **b** PBMNCs and **c** PCCs groups. *Legend*: RTW, return to work; PBMNCs, peripheral blood mononuclear cells; PCCs, purified CD34^+^ cells; AICLI, angiitis-induced critical limb ischemia

The eight dimensions of the SF-36 v2 was used to assess the QoL of patients at admission and 12 months, 36 months and 60 months after transplantation. In both groups, significant and persistent improvement in QoL was observed throughout the follow-up period (Fig. 6). Regarding RTW, 40 (18 in PCCs and 22 in PBMNCs) of the 50 AICLI patients returned to work during the 5-year follow-up. Most RTW patients (95.0%, 38/40) were re-employed within 12 months after transplantation and returned to the same job that they had preoperatively (Fig. 7B, C). Within 6 months, 17 PBMNCs patients and 11 PCCs patients returned to work; between 6 and 12 months, 5 PBMNCs patients and 6 PCCs patients returned; and between 12 and 24 months, 2 more PCCs patients returned. During the 5-year follow-up, 9 patients never returned to work, including 1 patient who was lost to follow-up, 1 patient (57 years old) retired early, 4 patients preferred not to work owing to the AICLI, and 3 patients could not work owing to the AICLI. The cumulative RTW rates gradually increased and peaked at 12 months in the PBMNCs group and 15 months in the PCCs group. The 5-year cumulative RTW rates were 88.0% in the PBMNCs group and 76.0% in the PCCs group, and the 2 groups did not differ with respect to the probability of posttransplantation RTW (log rank test, *P* = 0.085) (Fig. 7A).

## Discussion

The efficacy of stem cell transplantation, including PBMNCs, PCCs and bone marrow mononuclear cells (BMMNCs) transplantation, in promoting vasculogenesis and angiogenesis have been reported by many studies [11–15]. Compared with PBMNCs and PCCs, BMMNCs have similar long-term efficacy [5, 15] but are less feasible for use due to the tedious and risky steps required to obtain the cell product, including bone marrow aspiration and general anesthesia. Although PBMNCs and PCCs are widely used, comparisons between them have rarely been reported. The study we initiated in 2014 was the first randomized single-blinded parallel-group controlled trial (ClinicalTrials.gov: NCT 02089828) to compare the efficacy of PBMNCs and PCCs in patients with AICLI. The 3-year outcomes of the study showed the noninferiority of PCCs compared to PBMNCs with respect to MAFS and TAFS [8]. The 5-year results from the current study showed that there were no significant differences in MAFS or TAFS between patients who underwent PBMNCs transplantation and those who underwent PCCs transplantation, which confirmed and extended the results observed at 3 years.

Although the effectiveness and safety of cell therapy have been demonstrated, few studies have focused on the persistence of cell therapy. Recurrence is an indicator from which the durability of treatment is partly reflected. In the current study, among 5 TAO patients with recurrence, the mean recurrence time was 45.6 ± 16.2 months (range 21–66 months). Most (4/5) patients who developed recurrence failed to quit smoking after relief of ischemia, and the remaining 1 was also regularly exposed to secondhand smoke for work reasons. Exposure to tobacco is critical to the initiation, maintenance, and progression of TAO [16], and graft patency rates are nearly 50% lower in patients with TAO who continue to smoke after surgery [17]. From the results of the current study, we could conclude that both PBMNCs and PCCs transplantation have satisfactory outcomes in terms of ischemia relief and durability of transplanted limbs, with 5-year recurrence-free survival rates of 87.5% and 91.7%, respectively. New lesions represent the onset of new AICLI in the untreated limb and might reflect the systemic efficacy of cell transplantation to some extent. In the current study, PBMNCs patients seemed to be more likely to survive to the 5-year follow-up without developing new lesions than PCCs patients (100.0% vs. 83.3%, log rank test, *P* = 0.039). We speculate that this might result from the differences in the composition of the 2 types of cell products. After local intramuscular injection, a certain number of transplanted cells and/or the cytokines they secrete may enter the circulatory system and induce some systemic effects. Compared with PCCs, PBMNCs are characterized by a larger proportion of CD34^−^ cells, which could also assist in angiogenesis by secreting cytokines [18–20]. Recently, Bachelier et al. identified a novel CD34^−^133^+^ EPC subpopulation and found that it more potently mediates homing and vascular repair than CD34^+^ 133^+^ EPCs [21]. Therefore, this phenomenon might be induced by the weaker systemic angiogenesis effect of PCCs due to their lower proportion of CD34^−^ cells. In addition, compared with patients with recurrence, patients with new lesions seemed less likely to obtain relief from conservative treatment. In view of 2 out of 3 patients with new lesions who underwent second transplantations and the remaining 1 who underwent major amputation, more intensive treatment should be considered for patients with new lesions.

Considering that AICLI patients tend to be of younger age and male, RTW after transplantation is a health-related economic index of significant importance not only for the patients and their families but also for society as a whole. RTW not only demonstrates that patients’ physical health was sufficient for them to undertake work but also demonstrates their psychological well-being [22, 23] and is an important index for evaluating posttransplantation efficacy. In the current study, similar and satisfactory 5-year cumulative RTW rates were observed in both groups, and most RTW patients continued their preoperative work, demonstrating that satisfactory psychological and physical recovery could be achieved after cell therapy including PBMNCs and PCCs in AICLI patients. In particular, among 9 patients who never returned to work during the 5-year follow-up, there were only 3 patients who could not work owing to the AICLI. Additionally, 4 out of 9 patients who preferred not to work due to the AICLI became CLI-free during the 5-year follow-up.

From the current study and our previous studies [7, 8], most significant efficacy outcome improvements could be observed at 6 months, indicating that the short-term observation period might be adequate to determine the effects of cell transplantation. On the other hand, most recurrence/new lesion events (7/8) occurred 24 months posttransplantation, demonstrating the satisfactory and persistent mid-term efficacy of cell transplantation and suggesting that more attention should be given to the ischemic condition 2 years posttransplantation. The 5-year outcomes of this trial demonstrated similar safety and efficacy outcomes between the 2 autoimplants for limb salvage and ischemia relief. In terms of the 2 types of cells, as we reported previously [7, 8], PBMNCs were characterized by many advantages, including a larger absolute number of CD34^+^ cells (suitable for patients with multiple ischemic limbs needing transplantation), earlier RC improvement and higher cost-effectiveness. In the current study, PBMNCs patients were shown to be more likely to survive to the 5-year follow-up without new lesions. Therefore, from the long-term follow-up outcomes of the trial, we could conclude that PBMNCs transplantation is the preferred solution for AICLI patients.

There were some limitations in the current study. First, this was a single-arm study without a placebo-treated group. However, for patients who were in CLI condition after adequate conservative treatment, placebo use seemed to be unethical. Second, given the relatively small number of patients in our trial, the lack of power calculation cannot be ignored. Third, because only 3 enrolled patients had non-TAO-induced AICLI, trials with more non-TAO patients are needed to verify this conclusion in the future.

## Conclusion

In conclusion, the long-term follow-up outcomes of this trial not only further demonstrated similar efficacy and safety outcomes for the 2 types of autoimplants but also showed a satisfactory cumulative RTW rate in AICLI patients who underwent cell transplantation. Compared with PCCs patients, PBMNCs patients were more likely to survive for 5 years without new lesions. PBMNCs transplantation seemed to be the preferred solution for AICLI patients due to its many advantages. Validation of the conclusions is pending more evidence from a larger number of patients.

## Data Availability

Due to the confidential and identifiable nature of this dataset, data sharing will not be available. All authors have accessed the database and verified its accuracy.

## Abbreviations

AICLI: Angiitis-induced critical limb ischemia
PCCs: Purified CD34^+^ cells
PBMNCs: Peripheral blood mononuclear cells
MAFS: Major amputation-free survival
TAFS: Total amputation-free survival
WBFPS: Wong–Baker Faces Pain Rating Scale
PFWT: Pain-free walking time
ABI: Ankle-branchial index
TBI: Toe-branchial index
TcPO_2_: Transcutaneous oxygen pressure
